# Innovations in Hybrid Laparoscopic Surgery: Integrating Advanced Technologies for Multidisciplinary Cases

**DOI:** 10.7759/cureus.63219

**Published:** 2024-06-26

**Authors:** Muhammad Junaid Cheema, Muhammad Mustaneer Ul Hassan, Aiman Asim, Eemaz Nathaniel, Mohamed Ishraq Shafeeq, Muhammad Abbas Tayyab, Cijal Rahim Valiyakath, Shenouda Abdallah, Ali Usman

**Affiliations:** 1 General Surgery, Sir Ganga Ram Hospital, Lahore, PAK; 2 Medicine and Surgery, Jinnah Postgraduate Medical Centre, Karachi, PAK; 3 Research, Harvard Medical School, Boston, USA; 4 Surgery, Vitebsk State Medical University, Vitebsk, BLR; 5 Surgery, King Edward Medical University, Lahore, PAK; 6 Medicine, Guangxi Medical University, Nanning, CHN; 7 Surgery, Jaber Al-Ahmad Hospital, Kuwait, KWT; 8 General Surgery, Nishtar Medical University, Multan, PAK

**Keywords:** hybrid laparoscopic, prognosis, medicine, general surgery, surgery

## Abstract

Combining conventional laparoscopic techniques with cutting-edge technologies, such as robotics, improved imaging, and flexible equipment, hybrid laparoscopic techniques represent a revolutionary advancement in minimally invasive surgery. These methods have several benefits, such as increased accuracy, quicker healing periods, and fewer complications, which makes them especially useful in complicated multidisciplinary situations. The historical evolution, uses, benefits, and drawbacks of hybrid laparoscopic procedures are examined in this narrative review, which also covers urological, gastrointestinal, cardiothoracic, and gynecological surgery. The review focuses on how these methods promote interdisciplinary cooperation and creativity by enabling more accurate and successful surgical operations. It also discusses the equipment needs, integration difficulties, and technical difficulties that need to be resolved to reach the full potential of hybrid laparoscopic surgery. For hybrid laparoscopic procedures to become more widely used and effective in the future, there is a need for specialized training programs, interdisciplinary research collaborations, and ongoing technological advancements.

## Introduction and background

By enabling procedures to be performed through tiny incisions using a camera and specialist instruments, laparoscopic surgery, sometimes known as minimally invasive surgery, completely changed the field of surgery. Comparing laparoscopic procedures to conventional open surgery, which was first established in the late 20th century, laparoscopic procedures have considerably decreased postoperative discomfort, hospital stays, and recovery times [1]. Continuous technological developments, such as high-definition cameras, better instrumentation, and robotic-assisted systems, have enlarged the range and complexity of operations that may be performed laparoscopically and characterized the development of laparoscopic surgery [2].

Combining minimally invasive methods with the concepts of conventional open surgery, hybrid laparoscopic techniques seek to maximize the advantages of each. To handle complicated surgical problems that may be challenging with laparoscopy alone, these techniques frequently combine laparoscopic operations with other modalities, such as endoscopic or robotic approaches [3]. The value of hybrid approaches is found in their capacity to improve patient outcomes in complicated situations requiring a multidisciplinary approach, decrease invasiveness, and increase surgical precision [4].

This review primarily aims to investigate the use and effectiveness of hybrid laparoscopic methods in handling challenging interdisciplinary cases, specifically focusing on the integration of traditional laparoscopic techniques with advanced modalities such as robotic and endoscopic technologies. Through an analysis of various surgical specialties, we aim to provide a comprehensive understanding of how these combined approaches can be applied to maximize patient care. This review will detail the technical specifics of integrating basic laparoscopic techniques with advanced technologies such as robotics and endoscopy, highlight particular examples where such hybrid approaches have been successfully implemented, and assess the outcomes achieved.

A paradigm change in contemporary surgery, hybrid laparoscopic procedures provide creative answers to complex surgical issues. Surgical outcomes, operational timelines, and recovery rates may all be improved by combining the advantages of several surgical modalities. Knowing and using hybrid techniques can have a big impact on surgical practice by encouraging more effective and efficient patient care as healthcare systems give minimally invasive procedures more and more priority. This review attempts to emphasize the value of these methods and promote their wider use in the surgical community.

## Review

Hybrid laparoscopic techniques: an overview

Definition

Minimally invasive laparoscopic procedures and conventional open surgery are creatively combined in hybrid laparoscopic techniques. Using components of several surgical modalities, including robotic and endoscopic approaches, these procedures tackle difficult surgical problems that may not be possible with laparoscopic approaches alone. Using the advantages of minimally invasive techniques, such as less pain after surgery, shorter hospital stays, and faster recovery times, while preserving the adaptability and all-encompassing access of open surgery is the fundamental rationale behind hybrid laparoscopic surgery. Combining several surgical techniques in a sequence or simultaneously can improve the efficacy and safety of the operation [5].

Scope and Applications in Various Surgical Disciplines

Hybrid laparoscopic procedures, combining basic laparoscopic techniques with advanced modalities such as robotic and endoscopic systems, are highly flexible and adaptable, making them invaluable across several surgical specialties. These methods prove particularly effective when single-modality approaches are insufficient to address complex surgical challenges. Key applications in various surgical specialties are discussed below.

Gastrointestinal surgery: In the realm of complex gastrointestinal procedures such as colectomies (colonic resections) and proctectomies (rectal resections), as well as gastrectomies and esophagectomies, hybrid laparoscopic-endoscopic techniques are frequently employed. These advanced hybrid methods enable surgeons to perform intricate dissections, navigate challenging anatomical regions, and ensure complete oncological clearance with greater precision. The integration of laparoscopic and endoscopic approaches has demonstrated advantages over traditional methods, including shorter operative times, reduced complication rates, and faster recovery. Specifically, in the context of colorectal cancer, studies have documented improved oncological outcomes and decreased postoperative morbidity when these hybrid techniques are utilized [6].

Cardiothoracic surgery: Hybrid treatments for cardiothoracic conditions such as lung resections, mediastinal tumors, and complex coronary artery bypass grafting (CABG) combine open surgical techniques with minimally invasive thoracoscopic procedures. This integration enhances both surgical precision and patient outcomes. Research indicates that hybrid lung resections result in shorter hospital stays, reduced postoperative pain, and faster recovery compared to open thoracotomies. Hybrid CABG has been associated with improved cardiac function and reduced complication rates, offering a viable alternative for high-risk patients [7].

Urological surgery: Hybrid laparoscopic techniques are employed in urological procedures such as nephrectomies, prostatectomies, and cystectomies. These techniques enhance surgical precision and reduce invasiveness, leading to better patient outcomes. Robotic-assisted laparoscopic prostatectomy, for instance, combines the advantages of laparoscopy with the dexterity and visualization provided by robotic systems, enabling precise dissection and nerve-sparing techniques [8].

Gynecological surgery: Hybrid techniques have significantly advanced gynecological surgery, including hysterectomies, myomectomies, and endometriosis excisions. These methods often combine endoscopic guidance or robotic assistance with laparoscopic surgery, enhancing accuracy and reducing invasiveness. Robotic-assisted laparoscopic hysterectomies allow for precise dissection and suturing, reducing blood loss and postoperative pain compared to traditional methods [9].

Orthopedic surgery: Although less common, hybrid laparoscopic methods are gaining traction in orthopedic surgery, particularly for joint replacements and spinal disorders. Combining open and minimally invasive techniques improves surgical outcomes and shortens recovery times. In spinal surgery, for example, hybrid approaches utilizing robotic guidance enhance the accuracy of vertebral fusion, while in hip replacements, laparoscopic guidance combined with robotic assistance allows for precise implant placement [10].

The broad application of hybrid laparoscopic techniques underscores their potential to revolutionize surgical procedures across multiple disciplines. These methods represent a significant advancement in surgery by making procedures more precise, less invasive, and more effective.

Historical Development

Although laparoscopic surgery started in the early 1900s, it did not become widely accepted and popular until the 1980s and 1990s. The development of laparoscopic cholecystectomy in the late 1980s signaled a turning point by proving the viability and advantages of minimally invasive surgery. This achievement sparked interest and innovation in laparoscopic procedures across many surgical specialties. As the field evolved, surgeons encountered increasingly difficult cases that presented challenges for traditional laparoscopic techniques, such as limited access to some anatomical areas, issues with complex dissections, and the need for increased accuracy. Hybrid laparoscopic techniques were developed to overcome these limitations by combining the benefits of laparoscopic procedures with conventional open surgery [1].

Early innovations: The minimally invasive surgical era began in 1987 when Dr. Erich Mühe performed the first laparoscopic cholecystectomy. Compared to open surgery, this technique demonstrated the potential for less postoperative pain, shorter hospital stays, and quicker recovery. Beyond cholecystectomy, laparoscopic techniques quickly expanded to include gynecology, urology, and general surgery treatments in the 1990s. Surgeons using laparoscopic techniques for gynecological procedures, hernia repairs, and appendectomies laid the foundation for increasingly complex applications [11].

The emergence of hybrid techniques: Early in the new millennium, hybrid laparoscopic procedures emerged out of the necessity to enhance surgical outcomes in challenging patients. These methods combined or sequentially used laparoscopic instruments with additional modalities, including robotics, endoscopy, and advanced imaging technology, to increase surgical accuracy and efficacy [12]. The invention of robotically assisted surgical systems, notably the da Vinci Surgical System, in the early 2000s revolutionized minimally invasive surgery. With the enhanced dexterity, precision, and visualization these technologies offered, complex procedures that were challenging with traditional laparoscopy became more manageable. The combination of laparoscopy with endoscopic and robotic techniques facilitated more thorough and precise procedures in complex patients [13].

Technological advancements: Throughout the 2010s, technological advancements further propelled hybrid laparoscopic procedures. The introduction of advanced imaging technologies, such as 3D imaging and fluorescence-guided surgery, significantly enhanced the capabilities of hybrid techniques. These innovations improved intraoperative visualization and precision, enabling better surgical outcomes in challenging multidisciplinary cases. The latest development in this field is the integration of augmented reality (AR) and artificial intelligence (AI) in hybrid laparoscopic procedures. AR provides real-time, enhanced visualization of anatomical structures, while AI-driven systems assist with surgical planning and intraoperative decision-making, further pushing the boundaries of what can be achieved with hybrid techniques [14].

The evolution of laparoscopic methods into hybrid techniques over time reflects the continuous pursuit of innovation and improvement in surgical practice. By combining multiple modalities and leveraging technological advancements, hybrid laparoscopic procedures have revolutionized surgery and opened new avenues for managing complex multidisciplinary patients. This ongoing evolution underscores the commitment to enhancing surgical precision, reducing invasiveness, and improving patient outcomes across various medical disciplines.

Applications in complex multidisciplinary cases

Gastrointestinal Surgery

Hybrid laparoscopic procedures have been essential in gastrointestinal surgery for managing complex cases that require meticulous and comprehensive treatments. These approaches often combine robotic or endoscopic support with laparoscopic techniques to enhance surgical outcomes. For instance, in esophagectomy, hybrid techniques merge laparoscopic dissection with endoscopic mucosal resection to ensure the complete removal of esophageal tumors while preserving as much healthy tissue as possible. Similarly, in laparoscopic colorectal procedures, robotic assistance allows for greater dexterity and precision in performing anastomoses and complex dissections [4].

Case studies have demonstrated the effectiveness of hybrid approaches in gastrointestinal surgery. One notable example involves research on gastric cancer patients, where minimally invasive gastrectomies were performed using hybrid laparoscopic-endoscopic techniques. Compared to traditional open surgeries, these hybrid procedures resulted in shorter operative times, reduced complication rates, and faster recovery. Similarly, in colorectal cancer cases, hybrid strategies have been associated with improved oncological outcomes and lower postoperative morbidity [15].

The advantages of hybrid laparoscopic procedures in gastrointestinal surgery primarily include better patient outcomes, reduced invasiveness, and increased precision. These methods enable surgeons to perform intricate operations with greater accuracy, minimizing damage to surrounding tissues and shortening postoperative recovery and discomfort. Additionally, the integration of robotic assistance and advanced imaging technologies further enhances surgical precision and outcomes [16].

However, hybrid approaches do present certain challenges. These include the need for specialized skills and training, longer operative times due to the complexity of the procedures, and higher costs associated with advanced technologies [17]. Moreover, coordinating the use of multiple modalities in a single operation can be technically demanding and requires seamless collaboration among the surgical team [18]. By addressing these challenges and capitalizing on the benefits, hybrid laparoscopic procedures in gastrointestinal surgery can continue to evolve and improve, offering significant advantages in the management of complex surgical cases.

Cardiothoracic Surgery

Hybrid techniques are increasingly utilized in cardiothoracic surgery to address conditions such as coronary artery disease, mediastinal tumors, and lung resections. One notable hybrid approach for lung resections combines open surgical techniques with minimally invasive thoracoscopic surgery. This method reduces the invasiveness of the procedure while ensuring precise tumor removal. Another compelling example is hybrid CABG, which treats complex coronary artery disease by integrating conventional surgical bypass with percutaneous coronary intervention [19].

Hybrid techniques in cardiothoracic surgery have demonstrated promising results [20]. For instance, studies comparing hybrid lung resections to traditional open thoracotomies have shown shorter hospital stays, reduced postoperative pain, and quicker recovery times. Additionally, hybrid CABG has emerged as a viable alternative for high-risk patients who may not be suitable candidates for traditional CABG alone, offering improved cardiac function and a lower risk of complications [21].

The adoption of hybrid techniques in cardiothoracic surgery underscores their potential to enhance surgical precision and patient outcomes. By combining the strengths of open and minimally invasive approaches, these techniques provide a balanced solution that minimizes invasiveness while maximizing effectiveness. The success of hybrid techniques in this field highlights their role in advancing surgical practices and improving patient care for complex cardiothoracic conditions.

Urological Surgery

Hybrid laparoscopic techniques are employed in various urological procedures, including cystectomies, prostatectomies, and nephrectomies. These methods frequently incorporate robotic assistance to enhance the accuracy and efficacy of laparoscopic operations. For example, in robotic-assisted laparoscopic prostatectomy, the advantages of laparoscopy are combined with the increased dexterity and visualization provided by robotic systems. This integration allows for precise dissection and nerve-sparing techniques, leading to improved postoperative outcomes [22].

In cystectomies, hybrid techniques facilitate the removal of the bladder with a combination of laparoscopic and robotic approaches, offering better control and precision while minimizing invasiveness. Similarly, nephrectomies benefit from hybrid methods by enabling surgeons to perform complex kidney surgeries with greater accuracy and reduced risk of complications. The use of advanced imaging and robotic systems in these procedures enhances the surgeon’s ability to navigate intricate anatomical structures, ultimately improving patient outcomes [23].

The incorporation of hybrid techniques in urological surgery underscores their significant potential in refining surgical practices. By leveraging the strengths of both laparoscopic and robotic modalities, these techniques provide a balanced approach that maximizes precision, reduces invasiveness, and enhances overall surgical effectiveness. The success of hybrid laparoscopic techniques in urology highlights their crucial role in advancing the field and improving patient care for complex urological conditions [24].

Gynecological Surgery

Hybrid laparoscopic techniques have significantly transformed gynecological surgery, particularly in procedures such as hysterectomies, myomectomies, and endometriosis excisions. These methods often combine endoscopic guidance or robotic assistance with laparoscopic surgery to enhance accuracy and reduce invasiveness. For example, robotic-assisted laparoscopic hysterectomies allow for more precise dissection and suturing compared to conventional laparoscopic or open hysterectomies, resulting in lower blood loss and reduced postoperative pain [25].

In myomectomy, hybrid techniques enable the complete removal of fibroids while preserving the integrity of the uterus. This is achieved by combining laparoscopic removal of fibroids with robotic assistance, which enhances precision and control. Similarly, in endometriosis excisions, hybrid approaches allow for thorough treatment of the condition with minimal invasiveness, improving patient outcomes and reducing recovery times [26].

The integration of hybrid laparoscopic techniques in gynecological surgery underscores their potential to revolutionize surgical practices. By leveraging the advantages of both laparoscopic and robotic modalities, these techniques offer a balanced approach that maximizes precision, minimizes invasiveness, and enhances overall surgical effectiveness. The success of hybrid techniques in gynecology highlights their crucial role in advancing the field and improving patient care for complex gynecological conditions [27].

Other Specialties

Hybrid laparoscopic techniques are also being applied in other surgical fields such as orthopedic and neurological surgery. In orthopedic surgery, these techniques are used for procedures such as spinal fusion, hip replacements, and knee arthroscopies. For instance, hybrid approaches in spinal surgery combine minimally invasive laparoscopic techniques with robotic guidance to enhance the accuracy of vertebral fusion and reduce recovery times. In hip replacements, hybrid techniques involving laparoscopic guidance and robotic assistance allow for precise implant placement and improved joint function [28].

In neurological surgery, hybrid laparoscopic techniques are utilized for complex procedures such as brain tumor resections and spinal cord surgeries. These techniques often involve the use of advanced imaging technologies and robotic systems to improve surgical precision and minimize damage to surrounding neural tissues. For example, hybrid approaches in brain tumor surgery combine laparoscopic methods with intraoperative MRI guidance to ensure complete tumor removal while preserving critical brain functions [29].

Advantages and challenges of hybrid laparoscopic techniques

Advantages

Patients benefit directly from the notable improvement in surgical precision brought forth by hybrid laparoscopic procedures. Surgeons can accomplish more accuracy in difficult operations by fusing cutting-edge technologies such as robotics, endoscopy, and imaging systems with conventional laparoscopic techniques. With this accuracy, blood loss is reduced, adjacent tissues are damaged less, and diseased tissues are resected more completely. For example, hybrid approaches in cancer surgery allow for exact tumor margins, which raises the possibility of total tumor removal and lowers the chance of recurrence [30].

Reduction of postoperative problems and recovery durations is one of the main advantages of hybrid laparoscopic methods. Smaller incisions, less discomfort after surgery, and shorter hospital stays are the outcomes of these minimally invasive procedures. When compared to those undergoing conventional open procedures, patients usually heal more quickly and resume their regular activities. Robotic-assisted laparoscopic prostatectomies and hybrid CABG are two procedures where studies have demonstrated hybrid techniques lead to fewer complications, lower infection rates, and faster recovery [31].

Using hybrid laparoscopic procedures promotes multidisciplinary cooperation and creativity among surgical teams. Many times, these methods call for combining knowledge from several disciplines, including neurology, cardiothoracic surgery, urology, and general surgery. By working together, new surgical techniques are developed and best practices from many disciplines are shared. Using cutting-edge technology also promotes ongoing development and adaptation, expanding the possibilities of minimally invasive surgery [5].

Challenges

Hybrid laparoscopic procedures have a steep learning curve for surgeons and considerable technological complexity despite their benefits. Learning these methods calls for specific education and expertise in both robotic systems and intraoperative imaging as well as conventional laparoscopic surgery. Surgeons may need a long period and considerable practice to become proficient because of the steep learning slope. Furthermore, it can be technically difficult and needs exact teamwork to combine several modalities during a single process [32].

Hybrid laparoscopic procedures need sophisticated tools and resources, which can be prohibitive for many medical institutions. Purchasing and maintaining robotic systems, cutting-edge imaging equipment, and specialist laparoscopic instruments are expensive endeavors. Surgeons, anesthesiologists, and support staff are among the skilled professionals that these operations need, along with well-equipped operating rooms. Resource limitations may lead to smaller hospitals or those with tighter resources finding it difficult to apply these strategies [5].

Including hybrid laparoscopic techniques in current surgical procedures can be difficult. These sophisticated methods may need to be accommodated by changes to conventional surgical procedures and protocols, which calls for meticulous preparation and coordination. Surgeon teams used to traditional techniques could also be resistant to change. A smooth integration requires that every team member be taught and at ease with the new methods. Moreover, it is imperative to create uniform norms and standards for hybrid laparoscopic operations to guarantee uniform and secure procedures at many medical facilities [33].

Future directions and innovations

Technological Advances

Hybrid laparoscopic techniques are directly related to the creation and application of new technology. Robotic system improvements are always enhancing surgical control, dexterity, and precision. Next-generation robots with improved haptic feedback, machine learning algorithms, and AI can help surgeons perform complex maneuvers more easily and with more accuracy. In real-time, for example, AR overlays and AI-powered picture identification can highlight important anatomical components and possible risks during surgery [34].

A further interesting field is the development of less intrusive equipment with greater mobility and flexibility. Micro-robots, magnetically operated devices, and flexible endoscopes are just a few of the innovations that allow surgeons to reach difficult-to-reach places with little damage to nearby tissues. Furthermore improving the accuracy and safety of hybrid laparoscopic procedures is the real-time, high-resolution visualization of interior structures made possible by the incorporation of sophisticated imaging modalities, such as intraoperative MRI and CT scans [35].

Future Trends and Potential Breakthroughs

Looking ahead, several trends and potential breakthroughs are poised to shape the future of hybrid laparoscopic techniques. One such trend is the increasing use of telemedicine and remote surgery. With advancements in telecommunications and robotic systems, expert surgeons can perform or assist in complex procedures remotely, expanding access to specialized care and improving outcomes in underserved regions [36].

Another significant trend is the development of personalized and precision surgery. Advances in genomics, proteomics, and metabolomics, combined with AI and big data analytics, are paving the way for tailored surgical interventions based on individual patient profiles. This approach can optimize surgical planning, reduce complications, and enhance recovery by considering each patient’s unique characteristics and risk factors [37].

Training and Education

As hybrid laparoscopic techniques become more prevalent, there is a growing need for specialized training programs to equip surgeons with the necessary skills and knowledge. These programs should focus on the unique challenges and opportunities associated with hybrid techniques, including the use of advanced technologies, multidisciplinary collaboration, and complex procedural planning. Simulation-based training, using high-fidelity surgical simulators and virtual reality (VR) platforms, can provide a safe and effective environment for surgeons to practice and refine their skills before performing procedures on patients [38].

Incorporation of Hybrid Techniques in Surgical Education

Incorporating hybrid techniques into surgical education is essential to ensure that future generations of surgeons are proficient in these advanced methods. Medical schools and residency programs should integrate hybrid laparoscopic training into their curricula, emphasizing both theoretical knowledge and practical skills. This integration should include comprehensive modules on the principles and applications of hybrid techniques, the use of advanced technologies such as robotics and imaging systems, and the management of complex surgical cases [39].

Simulation-based training is a key component of this educational approach. High-fidelity surgical simulators and VR platforms can provide a safe and effective environment for students and residents to practice and refine their skills. These tools allow for the replication of complex surgical scenarios, enabling trainees to gain hands-on experience without risk to patients. Additionally, AR systems can offer real-time guidance and feedback, further enhancing the learning experience [40].

Continuing medical education (CME) programs and workshops also play a crucial role in keeping practicing surgeons updated with the latest developments and best practices in hybrid surgery. These programs should offer opportunities for surgeons to learn about new technologies, refine their techniques, and stay current with evolving standards of care. CME activities can include hands-on workshops, live surgical demonstrations, and interactive lectures by experts in the field [41].

Moreover, fostering a culture of lifelong learning and continuous improvement is vital for the successful integration of hybrid techniques into surgical practice. Surgeons should be encouraged to engage in ongoing professional development, participate in research, and contribute to the advancement of hybrid laparoscopic methods. Collaboration with interdisciplinary teams, including engineers, computer scientists, and biomedical researchers, can further enhance the educational experience and drive innovation in surgical techniques [42].

Research opportunities

Areas Requiring Further Investigation

Despite the advancements in hybrid laparoscopic procedures, further research is necessary to fully optimize their use and outcomes. One critical area of investigation is the long-term safety and efficacy of these procedures compared to conventional techniques. Large-scale, multicenter randomized controlled trials are needed to provide robust evidence on the benefits and potential risks of hybrid techniques across various surgical specialties. Such studies will help establish the clinical value and reliability of hybrid laparoscopic methods [40].

Another significant research area is the development of new technologies and methodologies to overcome existing limitations. For instance, improving the ergonomics of surgical instruments and enhancing the efficiency of teaching tools are vital for the continued evolution of hybrid laparoscopic surgery. Advanced ergonomic designs can reduce surgeon fatigue and improve precision during lengthy procedures, while innovative teaching tools, such as VR simulations and AR guidance, can provide more effective training for surgeons [43].

Additionally, investigating the cost-effectiveness and impact of hybrid laparoscopic procedures on healthcare systems is essential to support their widespread adoption. Understanding the economic implications, including initial investment, maintenance costs, and long-term savings from reduced complication rates and shorter hospital stays, will help healthcare providers make informed decisions about implementing these advanced techniques. Comprehensive cost-benefit analyses and health economic studies can provide valuable insights into the financial viability and potential benefits of hybrid laparoscopic surgery for both patients and healthcare systems [44].

Potential for Interdisciplinary Research Collaborations

The complexity and multidisciplinary nature of hybrid laparoscopic techniques offer significant potential for interdisciplinary research collaborations. Combining expertise from fields such as engineering, computer science, biomedical sciences, and clinical medicine can drive innovation and address the challenges associated with these techniques. Collaborative research initiatives can lead to the development of new technologies, improved surgical protocols, and better patient outcomes [45].

For instance, the integration of advanced imaging technologies and robotics in hybrid laparoscopic surgery requires the expertise of engineers and computer scientists to develop and refine these systems. Biomedical scientists contribute to understanding the biological implications and optimizing the use of these technologies, while clinical practitioners provide practical insights and validate the effectiveness of new approaches through clinical trials and patient care [46].

One area ripe for interdisciplinary collaboration is the enhancement of real-time imaging and visualization techniques during surgery. Innovations such as AR and AI can be integrated into hybrid laparoscopic systems to provide surgeons with enhanced guidance and decision-making support. AR can overlay critical anatomical information onto the surgeon’s field of view, while AI algorithms can analyze intraoperative data to predict outcomes and suggest optimal surgical maneuvers.

Furthermore, collaborations between clinical researchers and data scientists can lead to the development of predictive models that improve patient selection and surgical planning. By analyzing large datasets of surgical outcomes, researchers can identify patterns and factors that contribute to successful procedures, thereby refining patient eligibility criteria and tailoring surgical techniques to individual needs [47].

Interdisciplinary research collaborations also have the potential to address the educational and training needs associated with hybrid laparoscopic techniques. Developing comprehensive training programs that incorporate VR simulations and hands-on practice can ensure that surgeons are well-prepared to perform these complex procedures. These programs can be designed and evaluated through collaborative efforts between medical educators, VR developers, and clinical experts [48].

## Conclusions

A major development in the field of surgery, hybrid laparoscopic procedures provide previously unheard-of precision, shortened recovery periods, and improved results in a broad spectrum of intricate interdisciplinary cases. The combination of modern technology and conventional surgical expertise in these techniques creates a new age of minimally invasive operations that can be used in gastrointestinal, cardiothoracic, urological, and gynecological surgeries, among other medical specialties.

There have been significant advancements in both surgical practice and technology along the path from traditional laparoscopic techniques to hybrid procedures. Robotic systems, cutting-edge imaging modalities, and flexible equipment have revolutionized surgery, making minimally invasive operations that were previously thought to be too complicated or dangerous possible. Because surgeons now have to adjust to the complexity and demands of these cutting-edge procedures, this development emphasizes the need for interdisciplinary cooperation and ongoing education.
